# Antibody-Mediated Growth Inhibition of *Plasmodium falciparum*: Relationship to Age and Protection from Parasitemia in Kenyan Children and Adults

**DOI:** 10.1371/journal.pone.0003557

**Published:** 2008-10-29

**Authors:** Arlene E. Dent, Elke S. Bergmann-Leitner, Danny W. Wilson, Daniel J. Tisch, Rhonda Kimmel, John Vulule, Peter Odada Sumba, James G. Beeson, Evelina Angov, Ann M. Moormann, James W. Kazura

**Affiliations:** 1 Case Western Reserve University, Cleveland, Ohio, United States of America; 2 Walter Reed Army Institute of Research, Silver Spring, Maryland, United States of America; 3 Walter and Eliza Hall Institute of Medical Research, Parkville, Victoria, Australia; 4 Department of Medical Biology, University of Melbourne, Melbourne, Victoria, Australia; 5 Kenya Medical Research Institute, Kisumu, Kenya; London School of Hygiene & Tropical Medicine, United Kingdom

## Abstract

**Background:**

Antibodies that impair *Plasmodium falciparum* merozoite invasion and intraerythrocytic development are one of several mechanisms that mediate naturally acquired immunity to malaria. Attempts to correlate anti-malaria antibodies with risk of infection and morbidity have yielded inconsistent results. Growth inhibition assays (GIA) offer a convenient method to quantify functional antibody activity against blood stage malaria.

**Methods:**

A treatment-time-to-infection study was conducted over 12-weeks in a malaria holoendemic area of Kenya. Plasma collected from healthy individuals (98 children and 99 adults) before artemether-lumefantrine treatment was tested by GIA in three separate laboratories.

**Results:**

Median GIA levels varied with *P. falciparum* line (D10, 8.8%; 3D7, 34.9%; FVO, 51.4% inhibition). The magnitude of growth inhibition decreased with age in all *P. falciparum* lines tested with the highest median levels among children <4 years compared to adults (e.g. 3D7, 45.4% vs. 30.0% respectively, p = 0.0003). Time-to-infection measured by weekly blood smears was significantly associated with level of GIA controlling for age. Upper quartile inhibition activity was associated with less risk of infection compared to individuals with lower levels (e.g. 3D7, hazard ratio = 1.535, 95% CI = 1.012–2.329; p = 0.0438). Various GIA methodologies had little effect on measured parasite growth inhibition.

**Conclusion:**

Plasma antibody-mediated growth inhibition of blood stage *P. falciparum* decreases with age in residents of a malaria holoendemic area. Growth inhibition assay may be a useful surrogate of protection against infection when outcome is controlled for age.

## Introduction

Epidemiological evidence shows that people living in *Plasmodium falciparum* malaria holoendemic areas who experience repeated or chronic blood stage parasitemia develop clinical immunity with increasing age [1]. This naturally acquired immunity is in part due to antibodies elicited in response to infection since passive transfer of sera from clinically immune African adults to malaria-infected children decreases the level of blood stage malaria coincidental with reduced symptoms [2], [3]. The mechanisms by which such antibodies protect against parasitemia are complex and have been suggested to include i) inhibition of erythrocyte invasion and growth by antibodies directed against proteins expressed by merozoites and subsequent intraerythrocytic developmental stages of the parasite [4]; ii) antibody-dependent mononuclear cell cytokine-mediated inhibition of intraerythrocytic parasite growth directed by antibodies to a limited set of antigens [5], [6]; and iii) sequestration and phagocytosis of malaria-infected erythrocytes in the spleen mediated by antibodies to parasite antigens expressed on the erythrocyte surface [7]–[9]. Understanding the roles of anti-malaria antibodies is important to advance knowledge of the fundamental processes that underlie age-related acquired immunity since repeated exposure to blood stage malaria has different immunologic consequences compared to first or infrequent malaria infection [10]. In addition, reproducible in vitro assays of antibody-mediated malaria immunity are needed as surrogate endpoints to inform clinical trials of blood stage vaccines that are tested in malaria endemic populations [11]–[13].

Previous studies of naturally occurring immunity have relied primarily on serologic methods to measure antibodies to recombinant malaria protein vaccine candidates, infected erythrocytes, and parasite extract [14]–[22]. Observed inconsistencies and the poor predictive value of these serologic assays for malaria infection and morbidity may be related to the lack of comprehensive analysis of antibody responses to multiple blood stage antigens, many of which may not be included in the assays performed, and the likelihood that serology alone does not reflect the functional activity of such antibodies, e.g. recombinant proteins may have a conformation dissimilar to that of the native protein. Evaluating the broad repertoire of functional antibodies to blood stage malaria may also be useful in the future if attenuated whole blood stage parasites are considered as a strategy to develop a human malaria vaccine [23].

Growth inhibition assays (GIA) quantify the functional activity of antibodies directed against multiple blood stage antigens by measuring parasite growth in the presence of “immune” plasma compared to “non-immune” plasma. GIA have been used in vaccine development for merozoite antigens to assess the relationship of antibody responses after immunization to the time and level of parasitemia following challenge infection in monkeys [24]–[26]. Vaccine trials of Apical Membrane Antigen-1 (AMA-1) and Merozoite Surface Protein-1 (MSP-1) in malaria naïve human volunteers have elicited high antibody titers with increased parasite growth inhibitory antibody activity but have not been correlated with protection (Spring et al, manuscript in preparation, Bergmann-Leitner et al, manuscript in preparation). Studies of persons with naturally acquired malaria immunity have shown an inconsistent relationship between serologic and functional antibody responses [17], [27]. Blood stage antigen (AMA-1 and MSP-1) vaccine studies in malaria experienced individuals demonstrate variable serologic and functional antibody responses, depending on the antigen tested [13], [28], [29]. Vaccine efficacy as related to GIA has been observed in animal models but not yet in humans.

Logistical impediments to the use of GIA in large scale population-based field studies have included the limited volume of blood that can be obtained from research participants, particularly children and infants, and time consuming determination of parasite growth endpoints. These logistical issues have in large measure been overcome [30]–[34]. In this study we used several methodologies from different laboratories to examine whether GIA could be used as a surrogate of protection against blood stage infection in children and adults living in a malaria holoendemic area of western Kenya.

## Methods

### Study population and study design

After community information sessions explaining the objectives of the research to understand the immune mechanisms underlying acquired resistance, assent and informed consent for enrolment and participation in the study was obtained from 117 healthy individuals (98 children and 99 adults) who were residents of the village of Kanyewegi, Nyanza Province, Kenya. The study took place in July 2003, during a time of relatively high malaria transmission. Previous studies from this area have shown that anti-parasite and clinical immunity to malaria is acquired with increasing age such that adult levels are attained by 10–14 years [35]. The average age of children and adults participating in our study was 7.7 and 39.4 years, respectively. Participants were asymptomatic without fever and had normal age-adjusted hemoglobin levels. Venous blood samples were collected prior to directly observed consumption of age- and weight-appropriate 6-dose regimens of CoArtem® (Artemether/Lumefantrin) regardless of baseline infection status. At baseline, 72% of children and 48% of adults had parasites present on peripheral blood smears. Weekly finger prick blood samples were collected for 11 consecutive weeks to determine time to infection by blood smear. All of the individuals included in this analysis had negative blood smears by microscopy two weeks post-treatment. Ethical approval for human investigation was obtained from the Institutional Review Board at Case Western Reserve University, University Hospital of Cleveland and the Ethical Review Committee at the Kenya Medical Research Institute. Adults participants signed a consent form in English or Duhluo (the local language); parents or guardians signed in the case of minors <18 years.

### Blood smear examination

Thick and thin blood smears (BS) were prepared, fixed in 100% methanol, stained with 5% Giemsa solution and examined by light microscopy for infected erythrocytes as described previously [36].

### Growth Inhibition Assays

Selected assays were performed with all 197 plasma samples, and all assays were performed with an identical subset of 54 plasma samples in three different laboratories using their standard procedures. The subset of 54 plasma samples was chosen to evaluate MSP-1_42_ specific antibody responses and T cell responses as they related to age. The subset contained equal numbers of adults (average age 50 years) and children (average age 5.8 years), and both groups contained equal numbers of MSP-1_42_ responders and non-responders. Power calculations indicated a sample size of 12 for each group was needed resulting in the subset of 54 (Moormann, manuscript in preparation). Plasma volume was not a limiting factor as all individuals had venous blood draws with several mL of plasma collected. Plasma samples were tested individually. No Kenyan plasma samples were pooled for any assay. For ease of description, the methods are described by laboratory location: CWRU = Case Western Reserve University, WEHI = Walter and Eliza Hall Institute, WRAIR = Walter Reed Army Institute of Research. Table 1 summarizes the GIA assays performed with the subset of 54 samples.

**Table 1:** Summary of GIA methodologies performed with 54 Kenyan plasma samples.

Parasite line	# of growth cycles	Stage harvested	Method of parasitemia measurement	Plasma dialyzed	Location GIA performed
D10	1	Ring	FACS (HO)	No	CWRU
D10	1	Ring	FACS (SYBR)	Yes	WEHI
D10	2	Late Troph	FACS (GFP)	Yes	WEHI
3D7	1	Late Troph	pLDH	Yes	WRAIR
FVO	1	Late Troph	pLDH	Yes	WRAIR
3D7	2	Late Troph	FACS (EtBr)	Yes	WEHI

HO = Hoechst 33342, Troph = trophozoite, pLDH = parasite lactic dehydrogenase, SYBR = SYBR Green I, GFP = green fluorescent protein, EtBr = Ethidium Bromide. CWRU = Case Western Reserve University, WRAIR = Walter Reed Army Institute of Research, WEHI = Walter and Eliza Hall Institute

#### CWRU

D10 parasites (D10-PfM3' [37]) were utilized and plasma was not dialyzed. Ring-stage parasites were synchronized twice by sorbitol lysis (5% D-sorbitol (Sigma, St. Louis, MO)) and allowed to mature to late trophozoite/schizont stages. Parasites were cultured at 4% hematocrit in RPMI-1640 supplemented with 25 mg/mL HEPES, 2 mg/mL sodium bicarbonate, 0.5% Albumax II (Gibco, Grand Island, NY), 2.4 mM L-glutamine, 0.08 mg/ml gentamicin, and 0.2 mM hypoxanthine. Cultures were maintained at 37°C in an atmosphere of 5% CO_2_, 1% O_2_ and 94% N_2_. Parasites were adjusted to 0.5% infected red cells with a final 2% hematocrit, 1∶10 plasma dilution, and 100 µL final volume in 96-well flat-bottom microtiter plates. The cultures were incubated for 26 hours to allow for schizont rupture and merozoite invasion (monitored by microscopy to ensure full schizont rupture). 25 µL of resuspended cultures were removed, fixed with 0.25% gluteraldehyde in PBS for 45 minutes, and placed in 1 µg Hoechst 33342 (HO) stain (Molecular Probes, Eugene, OR) in 400 µL 1× PBS for >24 hours at 4°C [38], [39]). Stained cells were examined with a BD LSR II flow cytometer to collect data from a minimum of 5×10^4^ cells. Becton-Dickinson FACS Diva 5.01 was used to collect and FlowJo 8.5.1 to analyze cytometry data. Where indicated, 10× SYBR Green I (Molecular Probes, Eugene, OR) instead of HO was used to stain fixed cells. Parasitemia was recorded as the number of infected erythrocytes. The mean parasitemia for duplicate wells was used to determine growth inhibition calculated with the following equation: 100-(test plasma parasitemia/non-immune plasma parasitemia×100). Plasma samples from four North Americans who had never been exposed to malaria were pooled as the “non-immune” plasma controls.

#### WEHI

D10 (D10-PfM3' [37]) and 3D7 parasites were utilized. Test plasma was dialyzed against PBS using 50-kDa-molecular-weight cutoff dialysis tubes (2051; Chemicon, Temecula, CA) and concentrated to the original starting volume using centrifugal concentration tubes (100-kDa MWCO; Pall Corp, Ann Arbor, MI) [34], [40].

D10 parasites (D10-PfM3' [37]) were transfected to express a green fluorescent protein (GFP) to facilitate detection by flow cytometry (Wilson, Crabb, and Beeson, manuscript in preparation). Parasites were cultured and assays performed as previously described [34]. Briefly, a synchronous late trophozoite parasite suspension of 0.4% with a final 2% hematocrit was cultured with a final plasma dilution 1∶10 in a final volume of 100 µL using U-bottom 96-well plates. The assay was harvested at two time points, after one and two cycles of parasite growth. This was accomplished by incubating the cultures for 26 hours to allow for schizont rupture and merozoite invasion (ring stage, monitored by microscopy). Then 20 µL of resuspended cultures were removed, fixed with 0.25% gluteraldehyde for 45minutes, placed in 10× SYBR Green I stain, and stored at 4°C [41]. After an additional 24 hours of culture, 35 µL media were added to each well. After a total of 96 hours of culture (two cycles of growth, trophozoite stage) the cultures were resuspended and GFP was detected using a FaCSCalibur flow cytometer.

GIA utilizing 3D7 parasites was performed using a parasite suspension of 0.1% with a final 2% hematocrit was cultured with a final plasma dilution 1∶10 in a final volume of 100 µL using U-bottom 96-well plates. The cultures were incubated for a total of 96 hours of culture (2 cycles of growth until trophozoite stage, monitored by microscopy) at which time 3D7 parasite containing wells were stained with 100 µL of 10 µg/ml ethidium bromide (Bio-Rad, Hercules, CA) in PBS for 1 hour [34]. Parasitemia was assessed using a FaCSCalibur flow cytometer. The mean parasitemia for duplicate wells was used to determine growth inhibition calculated as described above.

#### WRAIR

3D7 and FVO parasites were utilized. Test plasma was dialyzed against PBS followed by RPMI using 12-kDa MWCO [42] and heat-inactivated. The GIA was performed as previously described [31], [32] with synchronized schizonts and a starting parasitemia of 0.3% in 384-well flat-bottom plates with a final hematocrit of 2% in a final volume of 20 µL. The assays were cultured for one cycle with harvest at late trophozoite stage (i.e., 40 hours for 3D7, 48 hours for FVO). Cells were harvested and parasite lactic dehydrogenase (pLDH) was measured and detected as previously described [43]. The mean parasitemia for triplicate wells was used to determine growth inhibition calculated using the following equation: {1−([OD_test plasma_−OD_RBC_]/[OD_non-immune serum_−OD_RBC_])}×100. Non-immune serum pool of >5 individual sera obtained from the Interstate Bloodbank Tennessee was used as “non-immune” controls.

### Statistical analyses

Adults were defined as >14 years of age. Continuous variables were compared using Mann-Whitney, Spearman's rho, and Kruskal-Wallis tests. Inter-assay comparison of results were evaluated using Wilcoxon signed rank test, Bland-Altman plots to describe the equivalence of paired results [44], and the McNemar test to assess the differences in the proportion of responders (defined as >15% growth inhibition) vs. non-responders in paired assays. Kaplan Meier and Cox proportional hazards regression were used to compare time to infection across groups stratified by upper quartile vs. lower quartiles GIA responders. The assumption of proportionality was tested using plots of hazard functions and residuals. Analyses were conducted using SAS Version 8.2 (Carey, NC) and GraphPad Prism 4.

## Results

### Plasma exhibits parasite specific growth inhibition in GIA

**Figure 1:**
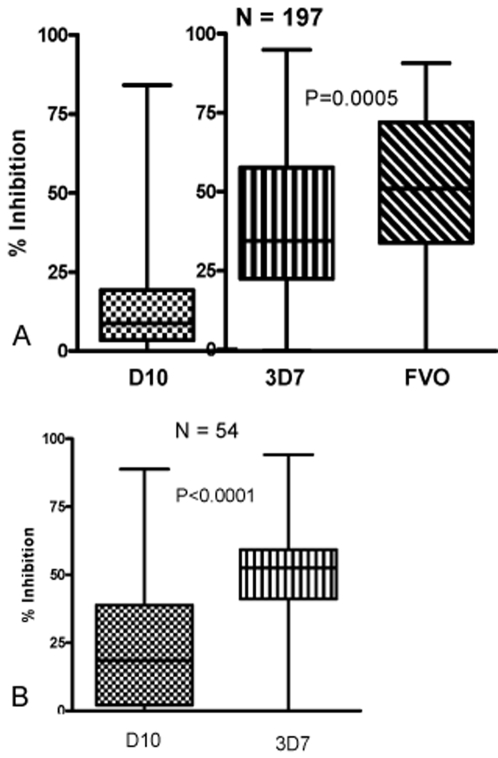
GIA of Kenyan plasma samples for various P. falciparum lines. Panel A, box plot of GIA result distribution for N = 197 samples for each parasite line tested (D10, 3D7, and FVO). Horizontal bars indicate the median percent growth inhibition. D10 GIA was conducted with different methodology than that of 3D7 and FVO GIA. All assays utilized one cycle of parasite growth and comparable methods of measuring parasitemia. Plasma exhibited higher growth inhibition against FVO than 3D7 (p = 0.0005, Mann-Whitney test). Panel B, box plot of GIA result distribution for N = 54 samples for D10 and 3D7 GIA utilizing the same methodology. Horizontal bars indicate the median percent growth inhibition. Plasma samples demonstrated more inhibitory activity against 3D7 than D10 (p<0.0001, Mann-Whitney).

**Figure 2:**
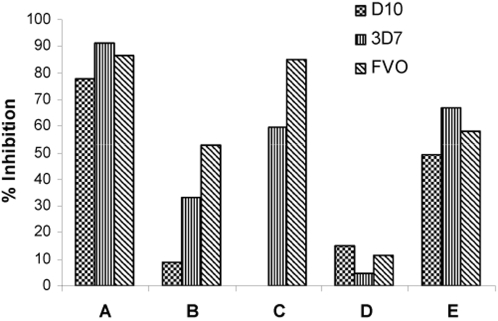
GIA of 5 representative Kenyan samples tested in parallel against 3 parasite lines (D10, 3D7 and FVO). Individual A has significant inhibitory activity against the 3 lines tested whereas individual D has essentially no inhibitory activity against all lines tested. Individual B has a growth inhibition pattern that reflects that of the entire population (Figure 1). Other individual samples demonstrate variable growth inhibitory activity against the different lines tested.

Plasma samples from 197 Kenyan adults and children were tested with various GIA against three different laboratory-adapted lines. The D10 GIA was performed at CWRU while the 3D7 and FVO GIA were performed at WRAIR. Plasma samples demonstrated more inhibitory activity against FVO (median 51.4%) than 3D7 (34.9%; p = 0.0005, Mann-Whitney test) as shown in Figure 1A. D10 GIA results are included in Figure 1A, but because assay methodology for D10 GIA was different from 3D7 and FVO GIA, direct comparison of results cannot be made. These assays utilized one cycle of parasite growth and comparable methods of measuring parasitemia were used. These plasma samples appear to have lower growth inhibition against D10 (median 8.8%) than 3D7 or FVO. Figure 1B demonstrates D10 and 3D7 GIA results with the subset of 54 plasma samples. These GIA were performed at WEHI using 2 cycles of parasite growth with parasitemia assessed by flow cytometry at the trophozoite stage. In these experiments, plasma samples demonstrated more inhibitory activity against 3D7 (median 52.6%) than D10 (median 18.5%; p<0.0001, Mann-Whitney). Growth inhibition levels are higher in these experiments compared to those conducted with all 197 plasma samples as the parasites were allowed to complete 2 growth cycles [34]. From these data we conclude that the same plasma samples exhibit different levels of growth inhibition with different parasite lines. Plasma samples exhibiting growth inhibition against 3D7 also tended to exhibit growth inhibition against FVO (Pearson correlation r^2^ = 0.763). The rank order magnitude of growth inhibition mediated against each parasite line was not consistent among individual plasma samples (Figure 2).

### Growth inhibitory activity decreases with age

**Figure 3:**
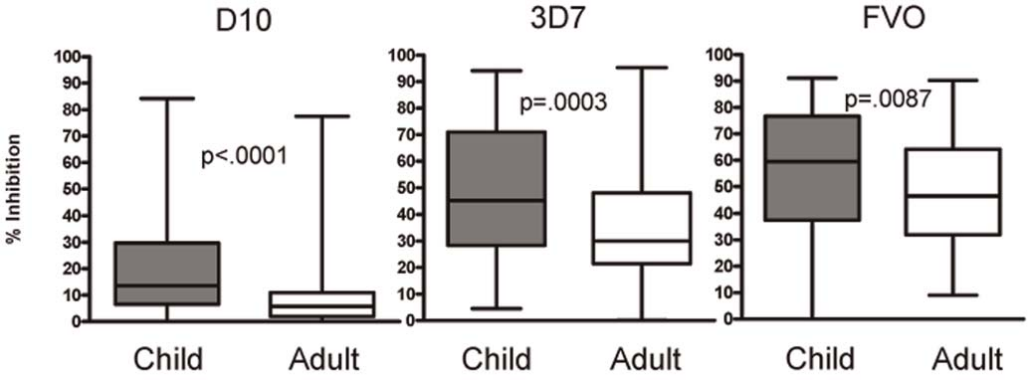
Box plot of GIA distribution for each parasite line tested (D10, 3D7, and FVO) divided by child (n = 98) and adult (n = 99). Horizontal bars indicate the median % growth inhibition. The child and adult growth inhibition medians differ significantly from each other for each parasite line tested (Mann-Whitney test). Child growth inhibition is consistently higher than adult growth inhibition for each parasite line tested.

**Figure 4:**
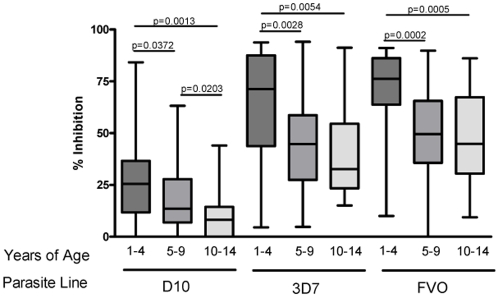
Box plot of GIA distribution for each parasite line tested (D10, 3D7, and FVO) with only children's plasma samples (n = 98) divided by age group: 1–4 years (n = 28), 5–9 years (n = 48), and 10–14 years (n = 22). The youngest age group consistently had higher growth inhibition compared to children in older age groups (Mann-Whitney test).

**Figure 5:**
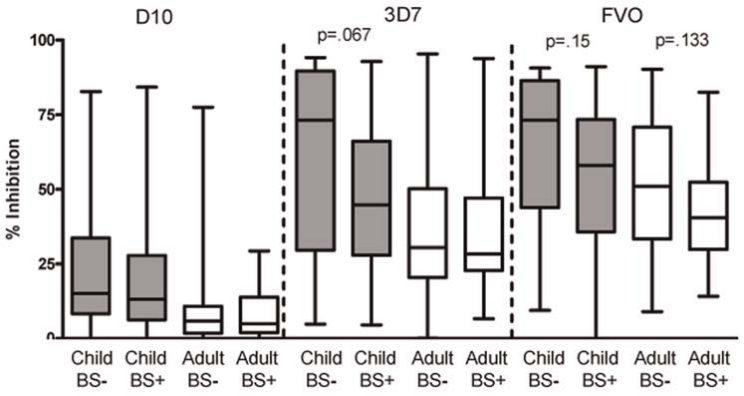
Box plot of GIA distribution of 197 plasma samples stratified by BS status at baseline and age for each parasite line examined (D10, 3D7, FVO). BS- children (n = 17), BS+ children (n = 81), BS− adults (n = 66) and BS+ adults (n = 33). No statistical difference was detected between any comparison groups (ie. BS− children vs. BS+ children with D10 GIA; Mann-Whitney test). A trend that BS− groups had higher growth inhibition than BS+ groups was observed. P values of visually dissimilar pairs are included.

The median level of growth inhibition was consistently greater for plasma from children than adults (Figure 3) regardless of parasite line, assay conditions and methods of measuring parasitemia (Mann-Whitney test: D10 p<0.0001, 3D7 p = 0.0003, and FVO p = 0.0087). To characterize the relationship between age and growth inhibitory activity, the 98 plasma samples from children were divided into three age groups; 1–4, 5–9, and 10–14 years. Note that the youngest child participating in this study was 15 months, and only two participants were less than 2 years. Thus, immune responses in children examined here were not affected by maternal antibodies which wane by age 9–12 months [45]. Growth inhibitory activity decreased progressively with each age group for all parasite lines tested (Figure 4). The youngest age group had significantly higher growth inhibition compared to the other age groups with all parasite lines tested. For D10 GIA, all age groups differed significantly from each other. For 3D7 and FVO GIA, the youngest age group differed significantly from the other two age groups. The 5–9 and 10–14 year old group GIA did not differ significantly from each other, though a decreasing trend was apparent. No significant difference was observed between the 10–14 year olds and adults. Additionally, baseline malaria infection status, controlling for age, did not affect GIA levels at a statistically significant level. However, a trend that baseline BS negative children and adults had higher GIA than baseline BS positive children and adults was seen especially in 3D7 and FVO GIA (Figure 5). A larger study population (we estimate at least n = 258 children and n = 3912 adults for 3D7 GIA) would be needed to observe a statistically significant difference in GIA stratified by baseline BS status.

### GIA is associated with increased time to malaria blood stage infection

Using a time-to-infection study design to examine the relation between growth inhibition *in vitro* and protection against blood stage infection, weekly finger stick blood samples were examined by microscopy for malaria parasites for the 11 weeks following administration of anti-malarial drugs. To accommodate the observation that the magnitude of growth inhibition differs among the various parasites lines (Figure 1), positive cut-off values were defined by the upper quartile of responders. Comparing high vs. low functional antibody assay responders has previously been used in Kaplan Meier analyses [46].

Time to infection was significantly associated with the degree of growth inhibition. While the time to infection differed between children and adults, the effect size (hazard ratios) were very similar (e.g., 3D7 GIA HR = 1.43, 95% CI = 0.85–2.38 for children and HR = 1.60, 95% CI = 0.78–3.28 for adults.). Therefore, age was treated as a confounder in these analyses as it was significantly associated with time-to-infection and growth inhibition (p<0.001).

Individuals whose plasma was within the upper quartile of growth inhibition experienced significantly less risk of infection compared to individuals with lower levels for the 3D7 GIA (hazard ratio = 1.535, 95% CI = 1.012–2.329; p = 0.0438) and FVO GIA (hazard ratio = 1.600, 95% CI = 1.014–2.526; p = 0.0435). These quartiles represented, respectively, positive growth inhibition cut-off values of 60% and 75%, corresponding to 53% and 60% less risk of infection.

**Figure 6:**
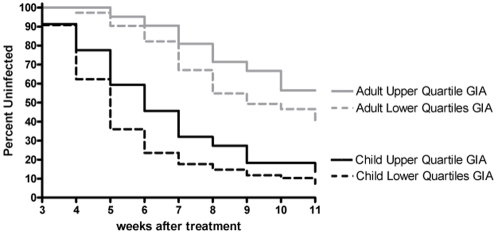
Relationship of growth inhibition with time to infection. Time to infection in individuals with upper quartile GIA results (>60% inhibition) tested against 3D7 compared to lower quartiles GIA results (<60% inhibition) controlling for age (Cox regression, p = 0.0438). Kaplan-Meier curves are divided by child and adult to illustrate age effects on growth inhibition. Similar analyses controlling for age were performed with D10 and FVO GIA.

Age remained an independent risk factor for infection in these proportional hazards models of GIA. For example, the magnitude of risk of infection decreased with each category of age from 1–4 years, 5–9, and 10–14, relative to adults (hazard ratio = 6.040 (3.719, 9.810); 4.160 (2.754–6.286); and 1.619 (0.897–2.923), respectively) controlling for 3D7 GIA. Figure 6 further illustrates that age is an essential feature of growth inhibition assessment. The hazard ratio was not significantly different for D10 at the upper quartile cutoff value (p = 0.25). However, a difference was observed among the 7% of individuals with the highest levels of growth inhibition using D10 (hazard ratio = 2.292, 95% CI = 1.009–5.208; p = 0.0475). Just as baseline infection status determined by positive BS had no effect on GIA levels controlling for age, baseline infection status did not affect time to infection controlling for age.

### Various GIA methodologies have little effect on parasite growth inhibition

Comparisons between GIA methods were undertaken to differentiate the effects of dialysis, culture media, heat-inactivation of plasma, flat vs. U-bottom tissue culture plates, number of growth cycles examined, and parasitemia measurement modalities. The same 54 plasma samples from the total 197 were examined with all assays. We focused on three main methodological disparities; 1) one vs. two growth cycles, 2) flow cytometry vs. pLDH assessment of parasitemia, and 3) dialysis vs. no dialysis of plasma. Method comparisons were made between GIA conducted with the same parasite line using the preferred methodology of the testing laboratory (summarized in Table 1). Inter-assay comparison of results were evaluated using Wilcoxon signed rank test, Bland-Altman plots to describe the equivalence of paired results [44], and the McNemar test to assess the differences in the proportion of responders (defined as >15% growth inhibition) vs. non-responders. Bland-Altman and McNemar tests have been shown to be superior to Spearman correlation tests (r^2^) when comparing assay results [31], [44].

**Figure 7:**
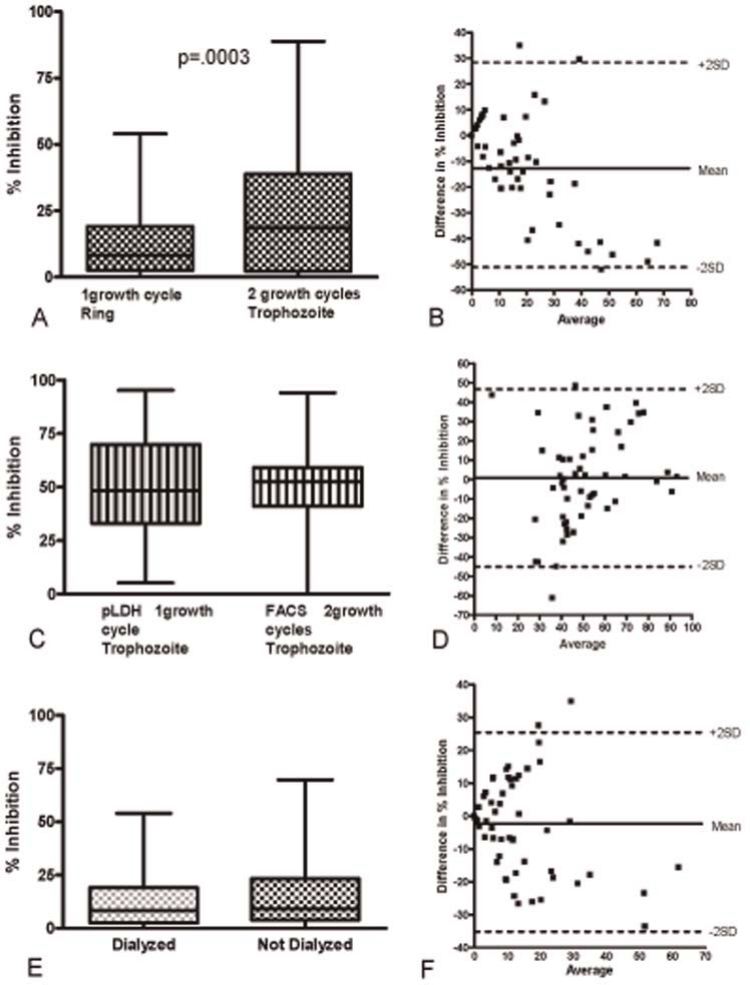
Comparison between different GIA methods. Panel A depicts box plots for D10 GIA using the same 54 dialyzed Kenyan plasma samples to compare one growth cycle (harvest at ring stage) to two growth cycles (harvest at trophozoite stage). Parasitemia was assessed by flow cytometry (10× SYBR Green 1 stain for one cycle, GFP for two cycles). Two growth cycles of D10 GIA had a statistically significant higher median inhibition (18.5% inhibition) compared to one cycle D10 GIA (8.3% inhibition; p = 0.0003; Wilcoxon signed-rank test). Panel B shows the Bland-Altman plot to assess the degree of agreement between paired results after one vs. two growth cycles. The points display the difference in growth inhibition between the two assays (y-axis) against their corresponding average values (x-axis). The horizontal lines correspond to the mean difference (solid line) ±2SD (dashed lines). The mean shows a bias of -11.05, indicating the two assays are producing different results with a trend of increasing differences with increased averages. Panel C depicts box plots for 3D7 GIA using the same 54 Kenyan samples. All samples were dialyzed prior to use. 3D7 GIA using one cycle of parasite growth, harvest at the trophozoite stage with parasitemia measured by pLDH was compared to 3D7 GIA using two growth cycles, harvest at the trophozoite stage with parasitemia measured by flow cytometry (EtBr). No statistically significant difference was noted (Wilcoxon signed-rank test). Panel D shows the Bland-Altman plot to assess the degree of agreement between paired assay results. The points display the difference in growth inhibition between the two assays (y-axis) against their corresponding average values (x-axis). The horizontal lines correspond to the mean difference (solid line) ±2SD (dashed lines). Panel E depicts box plots for D10 GIA using the same 54 Kenyan samples either dialyzed or not dialyzed. GIA was performed using one cycle of parasite growth (harvest at ring stage) followed by flow cytometry to measure parasitemia (SYBR Green 1 stain for dialyzed samples, Hoechst stain for non dialyzed samples). No statistically significant difference was noted (Wilcoxon signed-rank test). Panel F shows the Bland-Altman plot to assess the degree of agreement between paired assay results. The points display the difference in growth inhibition between the two assays (y-axis) against their corresponding average values (x-axis). The horizontal lines correspond to the mean difference (solid line) ±2SD (dashed lines). The mean has minimal bias (−2.8) with a small trend of increasing differences with increased averages.

GIA results were affected by the number of growth cycles parasites were permitted to complete (Figure 7A and B). Using D10 parasites and flow cytometry to measure parasitemia, growth inhibition was significantly greater with two cycles (96 hour culture, median inhibition 18.5%) compared to one cycle (24 hour culture, median inhibition 8.3%; p = 0.0003, Wilcoxon signed-rank test). A Bland-Altman plot shows a bias of −11.05, indicating the two assays produce different results. Also, the difference in the proportion of plasma donors classified as responders and non-responders in the two assays was statistically different (p = 0.006, McNemar test). The increased growth inhibition with two growth cycles may be attributed to amplified inhibitory antibody effects [34] and/or poorly characterized nutritional variables. Additionally, parasite growth from one to two cycles in the presence of human immune plasma could select for merozoite antigenic variation that may affect parasite invasion efficiency [40], [47]–[49] Thus comparison of different GIA must take cycle number into consideration.

GIA using 3D7 parasites performed with pLDH compared to FACS to assess parasitemia revealed no difference between the median growth inhibition levels for these two assays (pLDH, 48.4%; FACS, 52.6%; p = 0.88, Wilcoxon signed-rank test). Bland-Altman plots showed negligible bias (0.3), and the difference in proportions of responders and non-responders was not statistically different (p = 0.99, McNemar test) (Figure 7C and D). One important caveat is that pLDH was used to assess parasitemia after one cycle of growth (48 hour culture) whereas the FACS was used to assess parasitemia after two cycles (96 hour culture). Similarities in results of the two assays despite the shorter period of culture for the pLDH assay could be related to a wider range of responses for pLDH assay and therefore lower sensitivity compared to FACS [34]. We believe that flow cytometry using DNA stains [32], [38] is preferable to pLDH because of greater sensitivity [34] and to microscopy because of the capacity to evaluate greater numbers of infected erythrocytes with no observer bias.

Dialysis of plasma had no effect on the level of growth inhibition in this population of healthy asymptomatic Kenyans. GIA using FACS and D10 parasites at one growth cycle resulted in median growth inhibition of 8.3% and 9.1% for dialyzed and non-dialyzed plasma samples respectively (p = 0.165, Wilcoxon signed-rank test). Bland-Altman plots showed minimal bias (−2.8) and the difference in proportion of responders and non-responders was not statistically different (p = 0.40, McNemar test) (Figure 7E and F). Earlier studies confirmed that dialysis can effectively remove common antimalarials from plasma [34]. Others have attempted to avoid dialysis by using drug resistant parasites [50]. Indeed, the D10 PfM3' line used here contains a drug resistant cassette [37]. Since it is not possible to exclude definitively the presence of anti-malarial drugs or other non-antibody inhibitors, our recommendation is that standardized GIA include prior dialysis of plasma. Furthermore, well shape (flat vs. U-bottom), heat-inactivation of plasma, and different media components did not have an effect on growth inhibitory levels (data not shown). In sum, little difference in growth inhibition according to GIA method was appreciated beyond growth cycle number.

## Discussion

Antibodies that impair merozoite invasion and subsequent development of parasites in erythrocytes are one of several mechanisms by which persons who experience repeated malaria exposure during childhood are thought to acquire protection against high-density parasitemia and symptomatic infection later in life [4]. We compared in this study various GIA methodologies from three different laboratories and evaluated GIA results as potential immunologic surrogates of protection against blood stage infection in children and adults living in a malaria holoendemic area of Kenya.

An important and unexpected observation in our study was that growth inhibition decreased with age in all parasite lines tested, regardless of assay methodology. Indeed, the age-related decrease in growth inhibition parallels age-related decrease in the prevalence of high density parasitemia [35]. This inverse correlation between growth inhibition and age is counterintuitive when considered in the context of other studies showing that antibody prevalence and titers to blood stage antigens measured by serology increase with age [15], [51]–[57]. The data presented here thus underscore the notion that serologic and functional measurements of antibodies directed against merozoite pathways involved in invasion and intra-erythrocytic growth may be incongruent in persons with naturally acquired immunity, as demonstrated previously for antibodies to single antigens such as the C-terminal region of MSP-1 [46], [58]. It is not yet known why increasing age is associated with reduced overall growth inhibitory antibodies in our study population, but similar results are also reported by McCallum et al [59]. We speculate that single or few malaria infections may generate low-complexity “mono-specific” antibody responses directed against selected antigenic domains of merozoite ligands that are functionally critical to invasion, akin to the situation when malaria-naïve individuals are vaccinated with single candidate antigens [11], [12]. In contrast, the multiple infections experienced with increasing age in high malaria transmission areas may generate a more complex antibody repertoire, including a subset of antibodies that interfere with or impair functional inhibitory activity. Additionally, IgM antibodies that characterize early infections may have greater functional inhibitory activity than IgG antibodies which are predominant after greater numbers of infection. It is important to recognize that anti-malaria antibodies acquired as a result of natural infections can function in various ways, not only by preventing merozoite invasion and intra-erythrocytic growth as studied here, but also by binding to malaria proteins expressed on the erythrocyte surface and thereby facilitating phagocytosis and preventing cytoadhesion of infected erythrocytes [60], [61]. Furthermore, anti-idiotypic antibodies and immune complexes may develop with repeated infections and affect GIA levels. In any event, our data suggest that the mechanisms of protective immunity in children and adults may differ whereby antibody-mediated processes are more important in the former than the latter age group. Ongoing studies of MSP-1 specific immunity in Western Kenya are consistent with this since anti-MSP-1 antibodies appear to predominate in children whereas adults demonstrate more robust MSP-1 specific T cell immunity (Moormann, manuscript in preparation).

Comparison of GIA methodologies showed that inhibition was greater when parasites were allowed to undergo two rather than one growth cycle, but that measurements of parasitemia, dialysis of plasma and minor factors such as growth media and plate configuration did not substantially affect GIA results. On the other hand, the parasite line used in the GIA greatly affected the magnitude of plasma mediated growth inhibition. Differences in growth inhibition levels observed among 3D7, FVO and D10 are likely related to the high level of parasite antigenic diversity and the effect of natural selection on pathways involved in merozoite invasion and blood stage parasite development [62]. McCallum et al also report differences in the level of serum inhibition when using different laboratory adapted parasite lines or parasites that differ by invasion phenotype. Although our study and that conducted by McCallum et al utilized laboratory adapted parasite lines, differential growth inhibition by plasma against various field isolates has been demonstrated [63]. Using multiple laboratory and/or field isolates may improve the characterization of plasma growth inhibitory activity for a population.

Testing the hypothesis that GIA could be used as a marker of partial protection against blood stage malaria that develops in residents of malaria holoendemic areas, our results show that higher levels of growth inhibition activity were associated with a modest delay in time to infection with age as an important independent variable. Additionally, a trend was observed that individuals who were BS negative at baseline had higher GIA compared to those with positive BS, though this was not statistically significant. It is important to emphasize that GIA results likely represent only one of several immune endpoints that may be considered as surrogates of protection against blood stage malaria. It is possible that growth inhibitory antibodies have a more important function in children than adults, an important consideration in blood stage vaccine endpoint evaluation. GIA used in blood stage vaccine studies by the Malaria Vaccine Initiative (MVI) reference laboratory utilizes purified and concentrated IgG from plasma with one growth cycle parasitemia measured by pLDH [28], [43], [64]. Growth inhibitory activity of plasma is thought to be mediated primarily by IgG [34], [59]. Our use of diluted plasma in GIA may be comparable to MVI GIA, but our plasma samples were not tested by the MVI reference laboratory and therefore no definitive conclusions can be made. Understanding the development of parasite growth inhibition within the context of natural malaria infection and transmission is essential to blood stage vaccine endpoint evaluation. GIA would not be appropriate for use with pre-erythrocytic vaccine evaluation unless the targeted antigen were also highly expressed in the blood stage. Using GIA as method to predict the risk of malaria disease (our study had insufficient power for this analysis) and examination of other malaria-endemic populations is needed to corroborate our findings and further define the value of GIA as a tool to evaluate human malaria immunity.
